# Fully automated dual-resolution serial optical coherence tomography aimed at diffusion MRI validation in whole mouse brains

**DOI:** 10.1117/1.NPh.5.4.045004

**Published:** 2018-11-03

**Authors:** Joël Lefebvre, Patrick Delafontaine-Martel, Philippe Pouliot, Hélène Girouard, Maxime Descoteaux, Frédéric Lesage

**Affiliations:** a École Polytechnique de Montréal, Laboratoire d’imagerie optique et moléculaire, Montreal, Canada; b Université de Montréal, Research Centre, Montréal Heart Institute, Montreal, Canada; c Université de Montréal, Institut universitaire de gériatrie de Montréal, Montreal, Canada; d Université de Sherbrooke, Sherbrooke Connectivity Imaging Laboratory, Sherbrooke, Canada

**Keywords:** serial histology, diffusion MRI, optical coherence tomography, whole mouse brain imaging, multimodal registration

## Abstract

An automated dual-resolution serial optical coherence tomography (2R-SOCT) scanner is developed. The serial histology system combines a low-resolution (25  μm/voxel) 3× OCT with a high-resolution (1.5  μm/voxel) 40× OCT to acquire whole mouse brains at low resolution and to target specific regions of interest (ROIs) at high resolution. The 40× ROIs positions are selected either manually by the microscope operator or using an automated ROI positioning selection algorithm. Additionally, a multimodal and multiresolution registration pipeline is developed in order to align the 2R-SOCT data onto diffusion MRI (dMRI) data acquired in the same *ex vivo* mouse brains prior to automated histology. Using this imaging system, 3 whole mouse brains are imaged, and 250 high-resolution 40× three-dimensional ROIs are acquired. The capability of this system to perform multimodal imaging studies is demonstrated by labeling the ROIs using a mouse brain atlas and by categorizing the ROIs based on their associated dMRI measures. This reveals a good correspondence of the tissue microstructure imaged by the high-resolution OCT with various dMRI measures such as fractional anisotropy, number of fiber orientations, apparent fiber density, orientation dispersion, and intracellular volume fraction.

## 1 Introduction

Automated blockface histology is a maturing imaging technology that combines a tissue slicing apparatus, a motorized sample stage, and a microscope in order to image whole samples in three-dimensional (3-D) at high resolution. The common principle shared by all serial blockface histology methods is the repeated removal of small tissue layers to reveal new sample cross sections. This destructive imaging approach circumvents the limited penetration depth of light in tissue, and the motorized stage enables the acquisition of entire sample cross sections even with an imaging system using limited field-of-view (FOV) optics. Advantages of blockface imaging include the low quantity of sample deformation introduced by the tissue slicing process, its simple sample preparation protocol, and overall short acquisition time when compared to other serial histology methods such as serial histopathology^1^ or serial electron microscopy.^2^^,^^3^ In neuroimaging, automated blockface histology was reported with various optical modalities, ranging from confocal microscopy,^4^ fluorescence two-photon microscopy,^5^^–^^7^ CARS microscopy,^8^ optical coherence tomography (OCT),^9^ polarization sensitive OCT,^10^^–^^13^ and photoacoustic microscopy.^14^ Serial histology was used to study myelinated fibers,^15^^,^^16^ the neuronal connectome,^7^^,^^17^ the neurovasculature,^18^ Alzheimer’s disease,^19^ etc.

When designing a serial histology system or when performing an imaging study with such a system, a key aspect to consider is the imaging resolution. Using the tissue intrinsic contrast, high-resolution OCT can resolve fine tissue microstructure, such as the neuronal cell bodies^20^ and individual myelinated fibers^21^. Despite this advantage, high-resolution objectives are used at the expense of longer acquisition times, dataset size increase, as well as more complex and resource-intensive data reconstruction. For example, to acquire an entire mouse brain with a 40× objective offering sampling resolution of 1  μm over FOVs of $0.5\times 0.5\times 0.25\;{\mathrm{mm}}^{3}$ would require an estimated acquisition time of 60 days with our current serial OCT imaging system^9^ and would necessitate around 700 TeraBytes (TB) of disk space to store the dataset. On the other hand, low-resolution objectives offer the advantage of faster acquisition time and small dataset size at the expense of sacrificing tissue microstructure feature detectability. Nonetheless, low-resolution serial histology is sometimes desirable. In previous work, a serial OCT system^9^ was used with a 3× objective to perform whole mouse brain acquisitions at a resolution of 15  μm per voxel. The assembled SOCT mouse brains were then exploited to compute an average mouse brain template,^22^ which was coregistered to diffusion MRI (dMRI) brain data. The relatively poor resolution of the SOCT data did not allow for direct brain microstructure visualization, but the images are still useful in animal group studies^23^^–^^25^ and brain-wide investigation of the origin of OCT contrast in neuronal tissue. To take advantage of both the fast acquisition and reconstruction aspects of low-resolution serial histology and of the additional information provided by high-resolution OCT, a dual-resolution serial OCT scanner (2R-SOCT) was developed in this work. This system was then used in a multimodal study demonstration to compare the high-resolution OCT images acquired automatically with the 2R-SOCT imaging pipeline with dMRI data acquired in the same mouse brains prior to serial histology. Note that this paper is an extended and revised version of a conference proceeding^26^ presented at the SPIE Photonics West-BiOS conference in February 2018.

## 2 Methodology

### 2.1 Animal Sacrifice and Tissue Preparation

For this study, the Animal Research Ethics Committee of the Montréal Heart Institute approved all surgical procedures in accordance with the Canadian Council on Animal Care recommendations. A total of n=3 C57Bl/6 mouse brains were used. At sacrifice, the mice were anesthetized with 2% to 3% isoflurane and perfused transcardially with 20 ml phosphate buffered saline and then by a mixture of 4% paraformaldehyde (PFA) with 1% gadolinium (Gadovist) to reduce the T1 decay time and thus accelerate the dMRI acquisitions. Each mouse head was separated from its body and the skull was cleaned to remove muscles, lower jaw, vertebrae, and other tissues. The brain/skull was imaged in Fomblin with a multi-b-value high-angular resolution diffusion imaging (HARDI) MRI sequence described in the next section. After a dMRI acquisition, the brain was extracted from its skull and was embedded in a 4% agarose cylindrical block for serial imaging. All brains were positioned with the cerebellum facing up toward the microscope objective, such as the vibratome cuts were parallel to the brain’s coronal plane. The agarose gel was oxidized to create covalent cross links between the embedding medium and brain tissue, thus avoiding some cutting related artifacts such as tissue separation during the serial histology acquisition. A drawback of the oxidation procedure is that it renders the agarose brittle, which can cause structural damages while slicing with the vibratome. To avoid this effect, the 4% oxidized agarose cylindrical blocks were embedded in larger unoxidized agarose cylinders that provided better structural support. The agarose preparation and oxidation procedures followed the methodology presented by Ragan et al.^5^ In between tissue preparation steps and imaging sessions, the samples were kept in 4% PFA at 4°C.

### 2.2 Diffusion MRI Acquisition and Analysis

All fixed mouse brains were imaged prior to serial histology with a standard 3-D spin echo diffusion MRI sequence,^27^ using an Agilent 7-Tesla scanner equipped with 600 mT/m gradients and a custom-built 1-loop cylindrical coil (17-mm diameter, 20-mm long). The dMRI sequence parameters were: TE=0.022  s, TR=0.4  s, 70 gradient-encoding directions separated into 3 shells (6 directions with $b=400\;s/{\mathrm{mm}}^{2}$, 15 directions with $b=1066\;s/{\mathrm{mm}}^{2}$, 42 directions with $b=2000\;s/{\mathrm{mm}}^{2}$ and 7 interlaced acquisitions with b=0), δ=5  ms, Δ=12  ms, gradient amplitude 312.5 mT/m, FOV=16×12×8  mm, and an acquisition matrix of 128×96×64 giving an isotropic resolution of 125 μm, for a total acquisition time of 48 h. In order to obtain uniform angular coverage, the HARDI acquisition was designed using a generalization of electrostatic repulsion to multishell.^28^

The dMRI data preprocessing consisted in multiple registration, segmentation, and denoising steps as follows. First, all diffusion weighted images (DWI) and their associated b-vectors were coarsely rotated by π/2 angle increments until they were aligned with the neurological display convention. Then the first $b_{0}$ volumes of each DWI brains were extracted and used to compute an average anatomical brain template. This made use of the open source toolkit advanced normalization tools (ANTs^29^) and an iterative optimal shape and appearance template construction method.^30^ This optimal anatomical template was further aligned to the publicly available DSURQE T2-weighted *ex vivo* mouse brain template^31^ using rigid transformations. All DWI data were finally aligned to the anatomical template using rigid transformations, and the same rotations were applied to their associated b-vectors.

Most preprocessing algorithms and dMRI analysis performed in this study required brain extraction. This was performed using the “antsBrainExtraction” procedure provided in the ANTs toolkit and implemented in the Nipype neuroimaging data processing framework.^32^ The resulting brain masks were inspected in 3DSlicer^33^ to remove any segmentation errors. The remaining diffusion MRI preprocessing steps were performed to reduce noise and imaging artefacts.^34^ These correction steps included eddy current and sample bulk movement compensation,^35^ correction of the field homogeneity artefacts,^36^ reduction of the Rician noise bias,^37^ and an in-house time-varying signal bias compensation procedure, which is described in the Appendix.

Three dMRI analyses were performed on the data. First, in-house implementations^38^^,^^39^ of diffusion tensor imaging and HARDI reconstructions were performed using the Dipy library.^40^ Fractional anisotropy (FA) was computed from the local diffusion tensors with a non-negative least square method. The constrained spherical deconvolution with spherical harmonics (order 6) of Dipy^38^^,^^41^ was used to reconstruct the fiber orientation distribution functions (fODF). The principal directions of diffusion in each voxel and the maximum of the apparent fiber density (AFD_max)^42^^,^^43^ were extracted from the fODF. AFD_max is the maximal value of the fODF on the sphere, and it can be interpreted as the AFD_max. The number of fiber orientations (NuFO) within a voxel was computed with the method presented by Dell’Acqua et al.^39^ and using a data-driven threshold set to 1.5 times the AFD_max values in the ventricles. The last dMRI analysis performed was the neurite orientation dispersion and density imaging (NODDI) procedure.^44^ The AMICO python implementation^45^ of the NODDI model was used, with isotropic diffusivity $d_{\mathrm{iso}}=1.0\times 10^{-3}\;{\mathrm{mm}}^{2}/s$, longitudinal diffusivity $d_{∥}=0.6\times 10^{-3}\frac{{\mathrm{mm}}^{2}}{s}$, and regularization parameters $\lambda_{1}=0.5$ and $\lambda_{2}=1.0\times 10^{-3}$. Furthermore, the AMICO *ex vivo* option was used, which adds a fourth constrained water compartment to the NODDI model. This represents the water trapped by the tissue fixation. The choice of *ex vivo* diffusivity values for the isotropic and intracellular compartments was guided by previous values used in the literature.^44^^–^^46^ The NODDI fitting procedure resulted in two maps: orientation dispersion (OD) and the intracellular volume faction (IC_VF), which describes the water diffusion trapped within the axons and dendrites and that is usually interpreted as the neurite density.

### 2.3 Dual Resolution Swept-Source Serial OCT

**Fig. 1:**
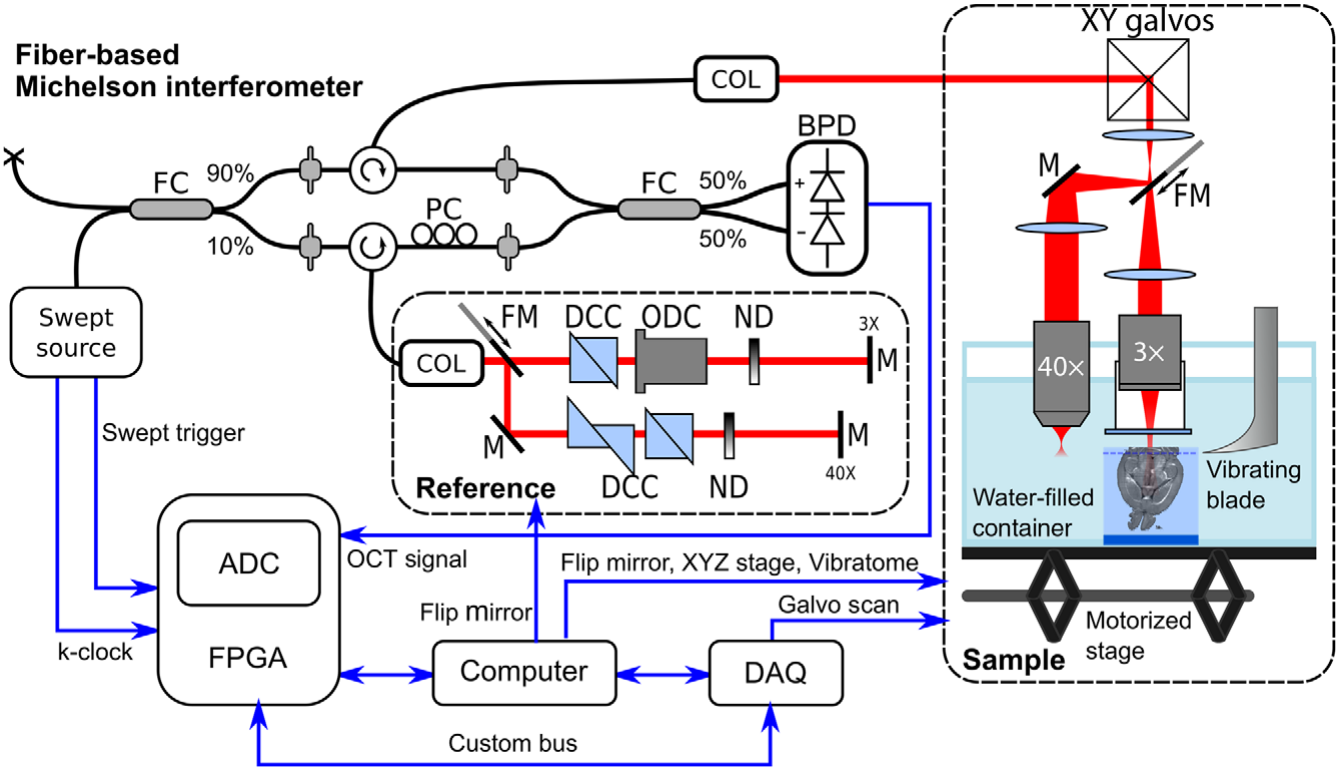
Main components of the dual-resolution serial OCT setup. FC, fiber coupler; COL, collimator; PC, polarization controller; BPD, balanced photodetector; FM, motorized flip mirror; DCC, dispersion compensation cube; ODC, objective dispersion compensator; ND, variable neutral density filter; M, mirror; ADC, analog-digital converter; FPGA, field-programmable array; and DAQ, data acquisition card.

A dual-resolution swept-source serial optical coherence tomography (2R-SOCT) microscope was developed (Fig. 1). This system is based on our previous single-resolution serial OCT design.^9^ The setup consists of three main components: (1) a fiber-based Michelson interferometer, (2) dual-resolution free-space sample and reference arms, and (3) an automated histology apparatus. The fiber-based Michelson interferometer input was a swept-source laser operated at a central wavelength of $\lambda_{0}=1310\;\mathrm{nm}$ with a tuning bandwidth of Δλ=100  nm (Axsun, 1310 Swept Source Engine). The swept-source laser generated a k-clock used to trigger the OCT volume scans and to acquire interference fringes, which are linearly distributed wavenumbers. The swept-source laser output was coupled to a 90/10 fiber coupler (Thorlabs, FC1310-70-10-APC); 90% of the laser power was targeted toward the sample arm and 10% to the reference arm. Single-mode fiber optical circulators (Thorlabs, CIR-1310-50-APC) were used in each arm to guide the laser output toward the sample and reference arms using fiber-optic collimators, and then to collect the output coming from each arm and send it toward the photodetector. A polarization controller (General Photonics, PolaRITE PLC) was used in the reference arm to adjust the contrast of the back reflected light interference fringes. The reference and sample signals were combined in a 50/50 fiber coupler (Thorlabs, FC1310-70-50-APC) and sent to a balanced photodetector (Thorlabs, PDB120C-AC). The interference fringes were recorded on a computer using a fast 12 bit waveform digitizer (Alazartech, model ATS9350, 500 MS/s).

The fiber-based Michelson interferometer outputs were connected to dual-resolution free-space sample and reference arms by fixed focus fiber collimators (Thorlabs, F280APC-C). The laser beam was scanned laterally with a small beam diameter galvanometer system (Thorlabs, GVS002). For low-resolution imaging configuration, the galvanometer mirrors’ output was sent directly to a 3× telecentric scanning lens (Thorlabs, LSM04 Scan Lens) using two optical lenses ($L_{1}$ and $L_{2,3X}$) in a telescope configuration. The 3× objective was enclosed in a custom-made watertight immersion chamber terminated by a wedged optical window (Thorlabs, WW11050-C). This immersion chamber had two purposes: (1) protect the scanning lens from the water and biological tissue debris created by the slicing process and (2) impose a constant air/water column in the sample arm. For high-resolution imaging configuration, a motorized flip mirror (Thorlabs, MFF101/M) was introduced between the first and second lenses of the 3× telescope. The laser beam was thus deviated into a second arm and a second telescope lens ($L_{2,40X}$), to end up in a 40× water-dipping microscope objective (Nikon, N40×-NIR). The telescope lenses focal lengths ($L_{1}=60\;\mathrm{mm}$, $L_{2,3X}=75\;\mathrm{mm}$, and $L_{2,40X}=125\;\mathrm{mm}$), the distance between the 3× and 40× arms (D=50  mm) and the immersion chamber height (H=40  mm) were optimized based on the target microscope objective aperture and on the axial distance between the 3× and 40× planes. As a result, the low-resolution objective focal plane was located 6.5 mm above the high-resolution focal plane. The free-space reference arm could be switched between 3×/40× arms in a similar manner using a motorized flip mirror. Dispersion compensation prisms (Thorlabs, PS908L-C) were located in each reference arm to physically compensate for optics induced light dispersion. Additionally, an objective specific dispersion compensator (Thorlabs, LSM04DC) was added to the 3× reference arm. Finally, variable neutral density filters were added to control the intensity of the measured interference fringes. The motorized flip mirrors were controlled directly by the acquisition computer via USB and the galvanometer system was controlled with a data acquisition card (National Instrument, NI-USB-6351).

The automated histology apparatus was the last component of this dual-resolution serial OCT system. The sample was placed in a water-filled acrylic glass container and was maintained in place using a custom-made 3-D printed sample holder. The focus depth position within the tissue was initialized by first manually adjusting the sample stage height until the tissue appeared in a live OCT B-scan. Then the focus depth was set automatically using the Fibonacci search method.^47^ The metric that was optimized was the average intensity within an A-line at the center of the lateral FOV. The Fibonacci search method was effectively aligning the 3× focal plane with the tissue surface. This automatic focus depth optimization was done only once at the beginning of the acquisition. Automated serial imaging was achieved by sequentially cutting thin tissue slices (around 200  μm) with a vibrating blade and by moving the sample under the microscope objective with a motorized stage (Zaber, T-LSR150B). At each motor position, the sampling beam was raster scanned over the objective FOV using galvanometric mirrors. An OCT A-line was acquired for each lateral sampling point, thus resulting in a mosaic of volumetric OCT tiles for each tissue slice. To reduce the total acquisition time, no A-line or volume averaging (spatial compounding) was performed. An overlap fraction of 20% was chosen between neighboring tiles. This overlap size was sufficiently large to always have enough tissue shared between neighboring tiles while limiting the quantity of data and increase in acquisition time when the overlap fraction is larger. After a slice acquisition, the sample was moved axially using a motorized jack (Thorlabs, L490MZ/ M), and this process was repeated until the whole tissue was sliced and imaged. The vibratome, XY stage, and Z labjack were all computer controlled using serial port communication.

Using python, the OCT volume reconstruction was achieved during the acquisition. After each OCT volumetric tile acquisition by the waveform digitizer, the data were transferred directly into memory. The reference interference fringe was computed from the data as the average fringe for a given tile, and this reference was then subtracted from the measured signal. To limit the side lobes introduced by the wavelength swept-source profile, a Gaussian apodization function [μ=1310  nm, σ=20  nm, which corresponds to a point-spread function (PSF) of FWHM=7.5  μm] was multiplied with each fringe. The A-lines at each raster scan position were obtained by computing the inverse Fourier transforms of the preprocessed signal. Only half of the axial range of the reconstructed volumes was recorded due to the Hermitian nature of real-valued signals. The volumes were recorded on a hard disk drive using the Nifti1 file format. In order to reduce the acquisition time, the high-resolution OCT volumes acquired with the 40× objective were reconstructed offline, i.e., after the serial histology acquisition.

### 2.4 Whole Brain OCT Volume Reconstruction

The data reconstruction method used to assemble the low-resolution OCT volumetric tiles into a single 3-D brain was presented in a previous publication.^9^ A key difference with our previous reconstruction model is that the precise tile positions were recorded during the acquisition for each volume, and these positions were used for the reconstruction instead of those given by registration. This modification accelerates the data acquisition and reconstruction procedures, and it provides a common reference frame for the dual-resolution acquisition paradigm described in the next section. Using the recorded XY tile positions, diffusion-based blending weights were computed for each overlap areas between neighboring tiles and were used to stitch the images together. Then the tissue attenuation coefficient was estimated from the data using a single-scattering model combined with the confocal axial PSF of the system. The extracted attenuation coefficients were used to compensate the depth-dependent OCT contrast in tissue. The axial translation between consecutive tissue slices was given by the labjack motor microstep position. Using these recorded positions, the slices were assembled into a single brain volume using 3-D diffusion-based blending weights. The tissue masks used for the whole brain reconstruction were optimized to ensure an overlap thickness of about 100  μm between consecutive slices. The whole brain reconstruction procedure was performed at an isotropic resolution of 25  μm per voxel.

### 2.5 Dual-Resolution Acquisition and Reconstruction

Two types of dual-resolution acquisitions were performed: (1) fixed focus 40× OCT mosaic acquisitions and (2) dynamic focusing 40× OCT acquisitions, also known as optical coherence microscopy (OCM). In order to define the regions of interest (ROIs) to be imaged, we developed a custom graphical user interface (GUI), which allowed the visualization of the last acquired brain slice as an average intensity projection (AIP) image. The displayed AIP, which was assembled during the acquisition, was located within the GUI viewport at its accurate Cartesian position given by the recorded motor positions. Using the GUI, any number of ROIs could be added by the microscope operator using either 0.5-mm width square ROIs for the OCM acquisitions or multiple connected line segments ROIs for the fixed focus mosaic acquisitions. The polygon defined by the closed-shaped polyline ROIs was converted into mosaics of 0.5-mm square tiles with 20% overlap fraction between adjacent tiles. Each ROI characteristics could be modified within the GUI, including the axial position of the first focal plane within the brain tissue, the axial thickness of the OCM acquisition, the spacing between consecutive OCT acquisitions, etc. For this study, the ROIs were defined manually using the GUI for one brain.

**Fig. 2:**
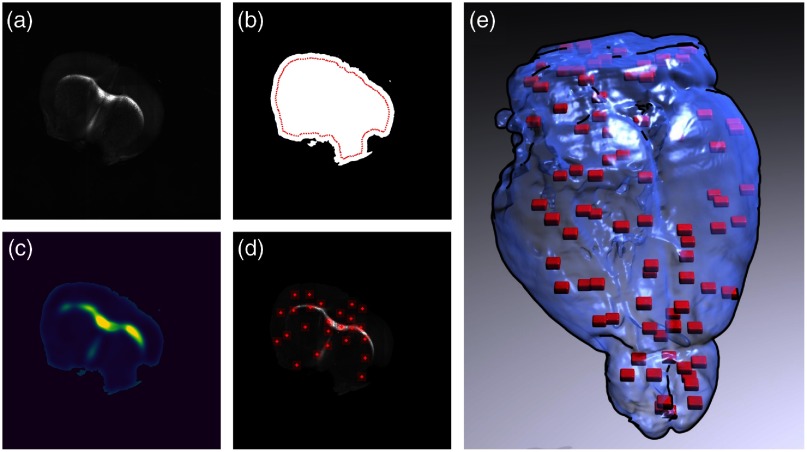
Automated 40× OCT ROIS generation method: (a) AIP of a 3× OCT mouse brain tissue slice, (b) tissue mask and 0.25-mm margin from the agarose/tissue boundary (red line), (c) probability bias used to guide the random ROI generation, (d) 25 ROIs of shape 0.5×0.5  mm generated for this slice, and (e) 3D rendering of the 40× ROIs (red) generated for an automated 2R-SOCT.

A second automatic ROI selection strategy, shown in Fig. 2, was employed for the two other brains. The ROI locations were generated randomly for a given slice by first computing a mask of the tissue within the AIP. The mask was computed using the tissue AIP by denoising the images with a bilateral filter and then by separating the pixels into two classes with the Otsu thresholding method. The resulting mask was denoised with a median filter and holes were filled with a morphological hole filling filter. The tissue mask was used to distinguish tissue pixels from agarose or water pixels. Then a two-dimensional (2-D) bias probability map was generated to guide the random ROI selection toward fibers and tissue areas exhibiting large contrast variations. The 2-D bias probability map was generated by summing the normalized tissue AIP with the normalized pixel intensity deviation from the average pixel intensity within the tissue mask. This image feature map was smoothed with a Gaussian filter of kernel size equals to 75  μm and was normalized by the sum of all features within the tissue mask. Low-probability values were representative of tissue areas with low-OCT reflectivity and where the intensity was close to the average tissue intensity. High-probability values were representative of tissue areas with high OCT reflectivity or where the intensity was either larger or lower than the average intensity within the tissue mask. To avoid acquiring images outside brain tissues, only ROI positions located within the segmented tissue and at least 250  μm from the agarose/tissue boundary were kept. Also a parameter was used to control the maximum overlap/minimum margin between any pairs of ROIs. To select the ROI positions based on the probability map, a list of valid ROI positions was generated from the tissue mask, and each position was associated with a probability as computed above. Then the “random.choice” method of the Numpy python package was used to select a position. The automatic ROI selection was performed every four slices. The whole automatic ROI selection algorithm, including 2-D stitching of the previous tissue slice acquired with the 3×-SOCT, tissue segmentation, probability map generation, and ROI position selection took 4 s per slice. The purpose of random ROIs selection was to show that the 2R-SOCT imaging pipeline can be fully automated, which results in reduced total acquisition time and also in less user-bias in the choice of ROIs.

Once all ROIs were selected for a given slice, the automatic dual-resolution acquisition began. For the OCM acquisitions, the sample was first moved to its 3× ROI Cartesian position and a small volume with the same 40× OCM ROI FOV was acquired. Then the sample was moved to its corresponding 40× position using the calibrated displacement between the 3× and 40× arms. The water/tissue interface was found automatically by acquiring multiple low sampling resolution 40× OCT volumes distanced axially by 50  μm and by detecting the axial position associated with the maximum value when convolved axially with the first derivative of a Gaussian. This was followed by an automatic focus depth fine-tuning using the same Fibonacci search method as for the serial histology focus depth initialization described for the 3× OCT. Once the water/tissue interface was located, the initial axial position was set and multiple 40× OCT volumes were acquired within the brain, separated axially by 16  μm. The interference fringe data were recorded on the computer for later offline OCM reconstructions. After the 40× dynamic focusing acquisition, the sample was moved back to the 3× arm, and the dual resolution acquisition continued with the other ROIs defined for this slice. The acquisition procedure for the dual-resolution fixed focus mosaics was similar, with the exception that multiple 40× OCT volumes were acquired at a single axial position and that the center of the mosaic was used to find the water/tissue interface for the whole dataset. The high-resolution OCM volumes were assembled from the sequences of OCT volumes acquired at multiple sample heights. The OCT volumes were blended together using the Gabor-based fusion methodology.^48^ The reconstruction was performed at a sampling resolution of 1.5  μm per pixel. Due to the small wavelength bandwidth of the swept-source laser, the axial resolution was much lower than the lateral resolution, limiting the subsequent OCM image analyses to 2-D *en face* planes only.

### 2.6 Multimodal and Multiresolution Registration

**Fig. 3:**
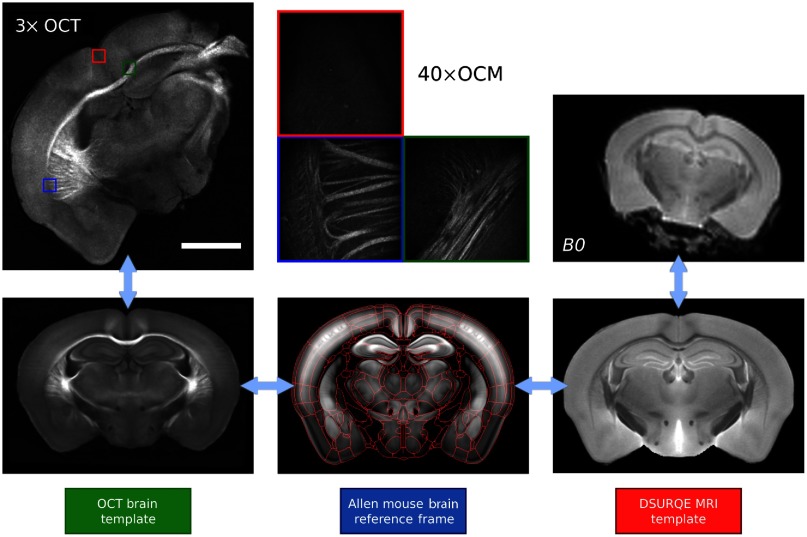
Multimodal and multiresolution coregistration workflow. In this illustration, all templates are shown at a resolution of 25  μm, the 3× OCT slice is at a resolution of 15  μm, the 40× OCM images are at a resolution of 1  μm and the dMRI $B_{0}$ map is shown at a resolution of 125  μm. The red lines shown in the Allen mouse brain template represent the brain structure boundaries as obtained from the Allen mouse brain atlas.

The precise alignment of the OCT and dMRI data is crucial in order to compare these imaging modalities. In previous works,^9^^,^^23^^,^^49^ we developed a multimodal registration procedure to map the assembled 3× OCT brains onto MRI images acquired on the same *ex vivo* samples prior to serial histology. This multimodal registration technique required intermediate registration templates for each imaging modality (dMRI and OCT) as represented in Fig. 3. Both registration templates were further aligned to the Allen mouse brain common coordinate framework^50^ (CCF) for brain structure identification. Each volume registration step was performed with a series of rigid and affine transformations and using the mutual information as a similarity metric. The ANTs tools were utilized to perform all registrations. The MRI registration template was the publicly available 40  μm DSURQE T2-weighted MRI *ex vivo* mouse brain atlas originally published by Dorr et al.^31^ from the Mouse Imaging Center (MICe). The first $b_{0}$ volume acquired during the dMRI session was used to perform the registration, and then transformations were applied to each b-value within the DWI. For the 3× OCT brain, the registration template was a 25-μm mouse brain template published previously by our group.^9^^,^^22^

### 2.7 Stereotactic Correspondence Between the 3× and 40× OCT

The precise positioning of the OCM volumes within the dMRI mouse brain was obtained by a combination of acquisition-related procedures and postacquisition registration techniques. First, the motorized sample stage was used to ensure an accurate and consistent correspondence of the 3× and 40× images. Although the linear stages employed for sample displacements possess submicron accuracy, the X and Y linear stages were not perfectly orthogonal, thus introducing a deformation when performing mosaics. To compensate this effect, a simple transformation model was developed to characterize the motorized sample stage assembly. This model describes the sample 2-D Cartesian positions as a combination of the linear stage microstep positions. The model was $\overset{\to }{p}=A{\overset{\to }{p}}_{M}+\overset{\to }{b}$, where A is a 2×2 transform matrix, $\overset{\to }{b}$ is a translation vector, $\overset{\to }{p}$ is the 2-D Cartesian position, and ${\overset{\to }{p}}_{M}$ is the microstep position of each linear stage. The transform matrix was calibrated for each linear stage by performing a displacement by predefined number of microsteps, by recording an image at each location and by then extracting the real translation performed between the initial image and the translated one using phase correlation. The linear stage origin location was taken into account by adding a translation $\overset{\to }{b}$. This model was used to precisely convert linear stage position to 2-D Cartesian positions. This accurate position was recorded for each 3× OCT tile, thus eliminating the need to perform pairwise image registration during the data reconstruction.

**Fig. 4:**
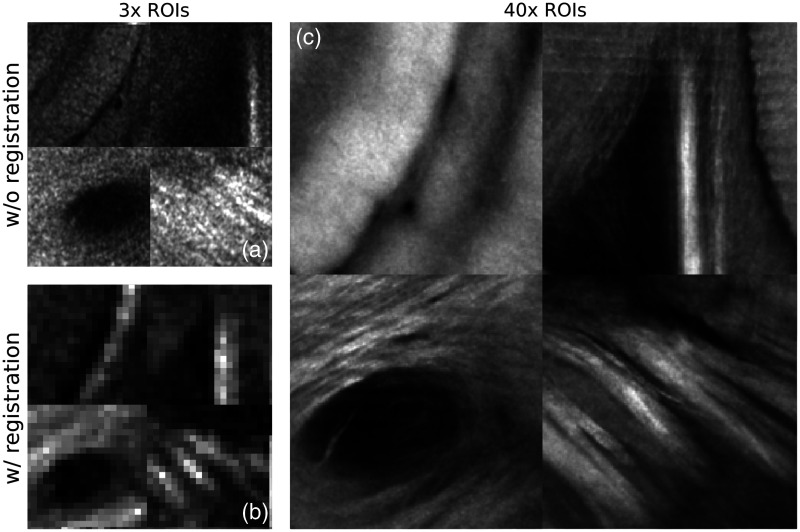
Example of 3× ROIs (a) without and (b) with registration and (c) their associated 40× ROIs. As shown by this example, the OCM to OCT registration is essential to find the accurate position of the 40× ROIs within the 3× OCT whole mouse brain.

Another calibration was performed in order to directly relate the images acquired by the 3× and 40× arms of the microscope. Using a resolution target, images of concentric circles were acquired using both the 3× and 40× objectives. The 2-D Cartesian coordinate corresponding to each image position was recorded, and the translation between both arms was measured. Consequently, images acquired with the 40× objective were mapped directly into the assembled 3× OCT brain slices during the 2R-SOCT acquisition process. A second offline registration procedure was employed to validate and refine the ROI positions for the subsequent multimodal comparison. A template matching algorithm (OpenCV) was used to find the lateral 40× ROI positions within an assembled 3× tissue slice. The similarity between the 40× templates and the 3× image patches was assessed with the normalized cross correlation.^51^ This registration procedure was necessary due to positional errors caused by the linear stages when performing large displacements to move the samples from the 3× to the 40× objectives (Fig. 4). Next, to be able to locate the 40× ROIs within the assembled 3× brains and the dMRI volumes, an ROI overlay volume was generated. The overlay generation method used the registered 40× lateral positions and the tissue slices positions computed for the 3× OCT whole brain reconstruction. The resulting overlay volumes had the same dimension as the assembled brains, and each ROI was represented by a 3-D block of similar FOV and position. Each block was assigned a different label to know which 3-D FOV corresponds to which 40× ROI. Finally, to get a stereotactic correspondence between the high-resolution 40× ROIs and their MRI counterparts, the affine matrices obtained during the 3× OCT and dMRI brain registration onto the Allen mouse brain were applied sequentially on the 40× ROI overlay volumes.

### 2.8 Multimodal Signal Comparison

**Fig. 5:**
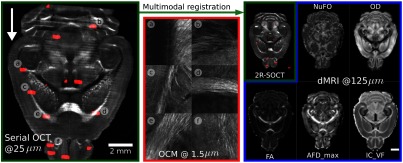
Multimodal signal comparison performed between the 2R-SOCT and the dMRI data. The 3× SOCT (green) is used to image a whole mouse brain that serves as a stereotactic reference to locate the 40× ROIs (red) within the dMRI volumes (blue). The OCT brain slice represented on the right (green inset) was coregistered to the dMRI data with a series of rigid and affine transforms. Scale bar: 2 mm.

The last part of this methodology was to compare the 40× OCM images with the dMRI data measured in the same brains prior to the automated histology (Fig. 5). The goal was to demonstrate the capability of the 2R-SOCT imaging pipeline to perform multimodal and multiresolution studies. First, the average dMRI measures associated with each 40× ROI was measured using the ROI overlay volumes that were registered to the MRI data. The average was performed over all the dMRI voxels encompassed by each 40× ROI. Second, the ROI overlay volume was coregistered to the Allen mouse brain CCF.^50^ All brain structures within the 40× ROIs were thus extracted from the Allen mouse brain atlas.^52^ The first ontological level in this atlas is separated into: (1) basic cell groups and regions, (2) fiber tracts, and (3) ventricular systems. For the purpose of this demonstration, all ROIs containing at least one structure classified within the “fiber tracts” ontological category were selected. Finally, this subset of the 40× ROIs was further separated based upon their dMRI measure average values. The ROIs were separated into four groups, using the 25%, 50%, and 75% quantiles of their associated dMRI measure as group discriminators. This was performed for the FA, the NuFO, the AFD_max, the NODDI, and the NODDI IC_VF. The 40× images classified with this multimodal and atlas-based selection criteria were finally inspected to assert if they exhibit brain structures commonly associated with low (<25% quantile) and high (>75% quantile) values of each of these dMRI measures. Finally, a few image features were computed within the 40× ROIs (average and standard deviation of reflectivity and attenuation). These features were categorized within each pair of low-/high-dMRI metrics and were compared in a quantitative way using Student’s t-tests.

## 3 Results

### 3.1 2R-SOCT Imaging System Characterization

The axial and lateral resolutions of the dual-resolution serial OCT system were measured using an USAF1951 resolution target (Thorlabs, R1L1S1N) and an OCT calibration phantom (Arden Photonics, APL-OP01). All measurements were done in water. The average lateral and axial resolutions of the 3× arms were $r_{xy}=17.5\;\mu m$ and $r_{z}=12\;\mu m$. The measured lateral resolution of the 40× arm was $r_{xy}=1.3\;\mu m$. The axial resolution of the 40× arm ($r_{z}=7\;\mu m$) was estimated by moving a mirror along the Z-direction and by measuring the FHWM of the intensity peak around the focus. To calibrate the automatic translation between the 3× and 40× arms, the concentric circles of the resolution target were used. The measured average radiant power at the sample was 9.4 mW, the OCT sensitivity was 98 dB, and the sensitivity roll-off was −0.2  dB/mm.

### 3.2 Dual-Resolution Acquisitions

**Fig. 6:**
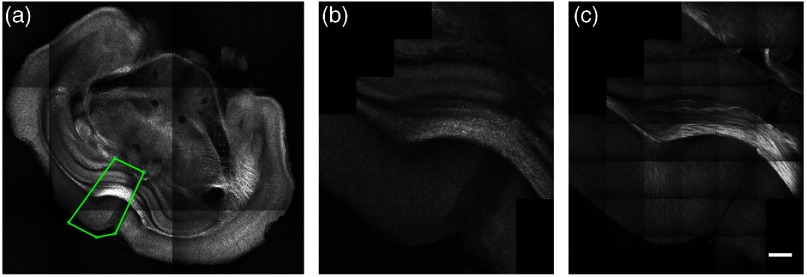
Example of a dual-resolution fixed-focus mosaic acquisition: (a) manual selection of the region of interest to be imaged (green polygon), (b) 3× OCT mosaic acquired at the defined ROI position, and (c) same region acquired with the 40× objective, assembled without preprocessing and blending. Each mosaic tiles have an FOV of $0.5\times 0.5\;{\mathrm{mm}}^{2}$ and an overlap of 20%. Scale bar: 250  μm.

An example of a fixed-focus dual-resolution mosaic acquisition is shown in Fig. 6. The ROI to be imaged was selected manually with a custom GUI using the AIP of the last acquired 3× OCT tissue slice [Fig. 6(a)]. Individual myelinated fibers appear in the neocortex, as shown in the high-resolution mosaic of Fig. 6(c). Also larger fiber bundles can be observed in the corpus callosum (cc). It is important to note that due to the orientation dependent OCT contrast of myelinated fibers,^9^^,^^20^ only the fibers that are orthogonal to the laser beam optical axis will appear bright in these images. Fibers that are parallel to the optical axis exhibit a dark contrast (e.g., the mammillothalamic tracts in the 3× mosaic or the cingulum bundle (cing) in the 40× mosaic of Fig. 6). Another source of OCT contrast in gray matter was hypothesized to be the neurite density, as defined by the ratio of neuronal cell bodies to neurites (myelinated axons and dendrites).^9^^,^^53^ This may be responsible for the visible structures in the neocortex or the hippocampus of the 40× mosaic. The ventricles and vessels appear in the *ex vivo* OCT images as dark areas. The vessels have a tubular or circular shape based on their orientation with the slicing plane. The microvasculature density could thus be another factor that affects the measured OCT contrast in *ex vivo* brain tissue.

**Fig. 7:**
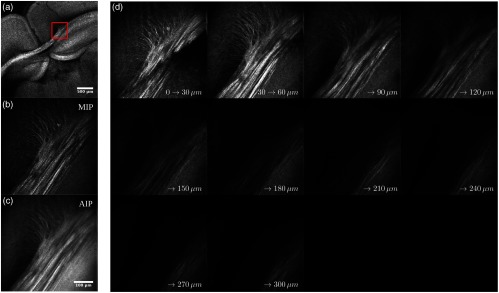
Dual resolution OCT acquisition showing the cc and cingulate bundle in a mouse brain: (a) low-resolution OCT volume used to target the high-resolution ROI (red rectangle), (b) maximum intensity projection, (c) AIP of the high-resolution OCM volume acquired, and (d) AIPs over 30  μm of the OCM volume. The scale bars are of size 0.5 mm for the 3× image and 100  μm for the 40× image.

An example of the second type of dual resolution acquisition (dynamic-focusing OCT or OCM) is shown in Fig. 7 for the cc. This brain area illustrates fiber bundle characteristics affecting the water diffusion signal analysis in dMRI, such as fiber OD, fiber crossing, and heterogeneity of fiber density and sizes. All OCM acquisitions exhibited high-signal attenuation and a degradation of the lateral PSF with depth. Each OCM acquisition took around 6 min to perform all movements, acquire a 3× version of the ROI, find the water-tissue interface, and acquire the 40× OCT dataset as raw interference fringe volumes. The offline OCM volume reconstruction took another 5 min per ROI. For the first brain in this study, the OCM ROI positions were selected manually, and due to the long acquisition time per dual-resolution position, the number of ROIs was limited to 4 to 5 areas every 4 to 5 slices, which extended the acquisition time to about 3 to 4 days.

**Fig. 8:**
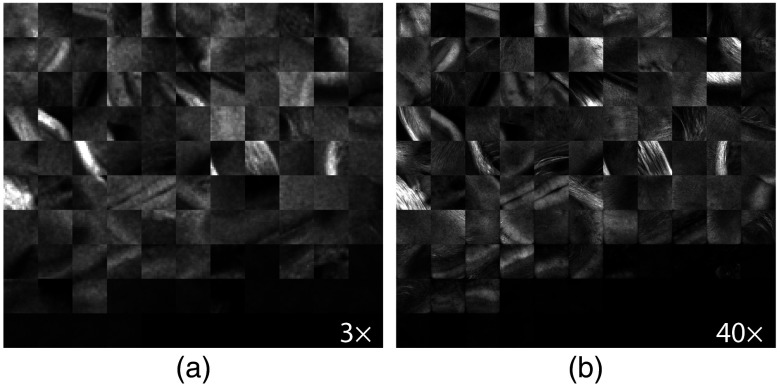
AIPs of the dual-resolution ROIs acquired automatically within a single mouse brain. (a) OCT ROIs acquired with the low-resolution 3× objective and (b) the same OCM ROIs acquired with the high-resolution 40× objective. Each ROI is of size $0.5\times 0.5\;{\mathrm{mm}}^{2}$.

For the second type of dual-resolution acquisition, the ROI positions could either be selected using the same GUI as for the dual-resolution mosaic images, or they could be generated automatically from the last acquired 3× OCT tissue slice. Figure 8 shows the AIPs of all dual-resolution ROIs acquired within a single mouse brain and using the automatic ROI selection method. Visual inspection of the ROI AIPs reveals that the 2R-SOCT system and template matching algorithm have successfully associated the 40× OCM volumes to their 3× OCT locations in each mouse brain slices. The difference between a few 3×/40× image pairs is mostly due to an axial range difference between the 3× and 40× AIPs. Indeed, the 3× OCT volume contains tissue from up to 800-μm deep, as of the 40× OCM volume axial range is going from the axial position of the water–tissue interface up to 250-μm deep. To characterize the ROI positioning accuracy and repeatability of the 2R-SOCT imaging system, a template matching registration was performed using as reference the position chosen by the microscope operator or the position generated by the automated ROI position selection algorithm. Average lateral shifts of Δx=24±31  μm and Δy=142±72  μm (mean ± std) were measured between the reference and registered ROIs positions. As the template matching was performed at the down sampled 3× OCT mosaic image resolution of 25  μm/pixel, the measured position shift in the X-direction corresponds to 1 to 2 pixels; thus part of the shift can be explained by image discretization. The larger lateral shift in the Y-direction could be caused by the way the sample stage is assembled. Indeed, the Y-axis linear stage is mounted on top of the X-axis linear stage and is only supported at its center, which acts as a pivot point. When performing a translation between the 3× and 40× objectives, the weight load is transferred from one side to the other of the pivot point. This can introduce minute linear stage displacements that affect principally the y-axis positions.

### 3.3 Comparison Between the dMRI and 2R-SOCT

**Fig. 9:**
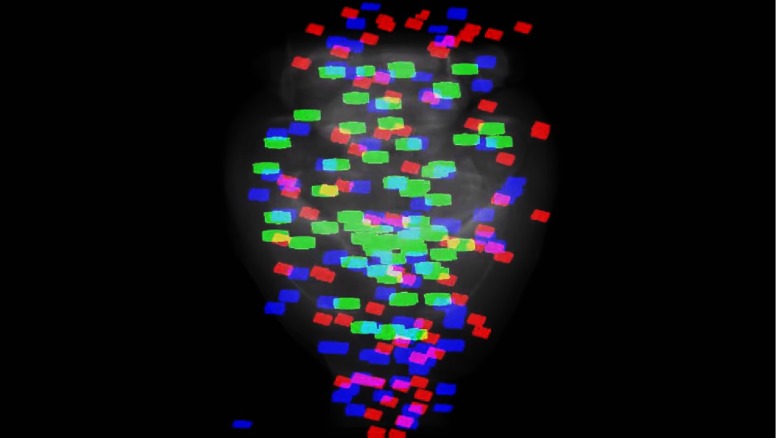
3-D rendering of all 40× ROIs of this study from three 2R-SOCT mouse brain acquisitions. All ROI overlay volumes were aligned to an OCT mouse brain template, shown here as a grayscale average intensity. The ROI position of the blue and red blocks was selected automatically by the 2R-SOCT ROI selection method, and the green blocks were selected manually by the microscope operator. This is a still frame from the video. (Video 1, MP4, 2.2 MB[URL: https://doi.org/10.1117/1.NPH.5.4.045004.1]).).

A multimodal comparison was performed between the OCM ROIs and the dMRI data. A total of n=250 ROIs were acquired across three brains. For one brain, the ROI positions were chosen manually during the acquisition by the microscope operator, and for the two other mouse brains, the ROI positions were selected automatically with the method presented above. A 3-D rendering of all ROI 3-D FOVs aligned to the Allen mouse brain template is presented in Video 1 (Fig. 9). As seen in this video, a few ROIs were located outside of the brain, mostly toward the anterior part. This is due to a tissue segmentation error when imaging near the olfactory bulb, thus ROI positions were generated in agarose instead of in the tissue. This problem could be addressed with improved tissue segmentation approaches. Furthermore, after the OCT brain registration onto the Allen mouse brain template, a few ROIs that were acquired in the medulla were outside of the FOV and were thus ignored in the multimodal comparison. Using the Allen mouse brain template, a subset of n=114 ROIs was selected among all acquired OCM volumes as they intersected at least one fiber tract structure.

**Fig. 10:**
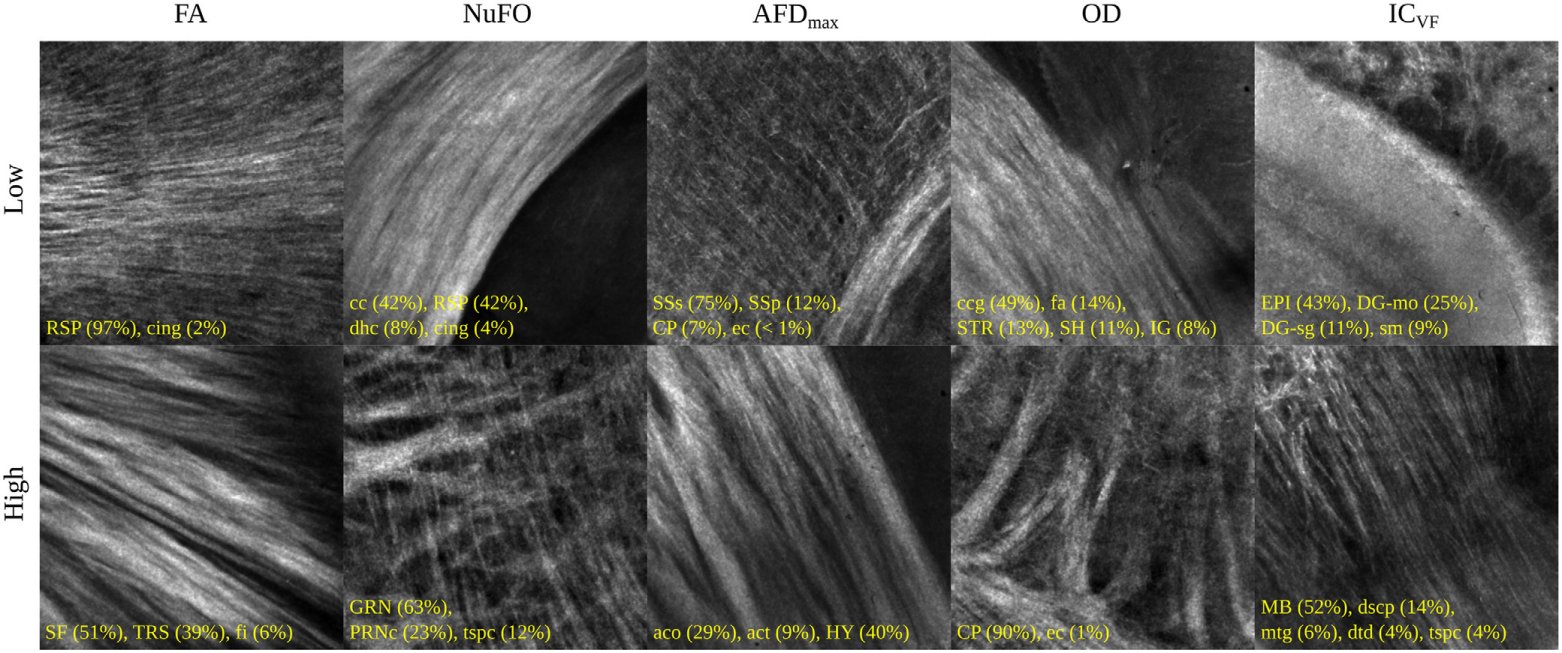
Examples of 40× ROIs associated with low- and high-dMRI measure values. Each image spans an FOV of $0.5\times 0.5\;{\mathrm{mm}}^{2}$ and is an AIP over 250  μm. The yellow annotations indicate the brain structures and their volume fraction within the ROIs. The acronyms follow the Allen mouse brain convention. RSP, retrosplenial area; cing, cingulum bundle; SF, septofimbrial nucleus; TRS, triangular nucleus of septum; fi, fimbria; cc, corpus callosum; dhc, dorsal hippocampal commissure; GRN, gigantocellular reticular nucleus; PRNc, pontine reticular nucleus caudal part; tspc, crossed tectospinal pathway; SSs, supplemental somatosensory area; SSp, primary somatosensory area; CP, caudoputamen; ec, external capsule; aco, anterior commissure olfactory limb; act, anterior commissure temporal limb; HY hypothalamus; ccg, genu of the corpus callosum; fa, corpus callosum anterior forceps; STR: striatum; SH, septohippocampal nucleus; IG, induseum griseum; EPI, epithalamus; DG-mo, dentate gyrus-molecular layer; DB-sg, dentate gyrus-granule cell layer; sm stria medullaris; MB, midbrain; dscp, superior cerebellar peduncle decussation; mtg, mammilotegmental tract; dtd, doral tegmental decussation; and tspc, crossed tectospinal pathway.

For this demonstration, 40× images associated with low- and high-dMRI measure values are shown in Fig. 10 for selected examples. The brain structures obtained with the multimodal and atlas-based ROI selection criteria exhibit characteristics that are in accordance with each measure value. All 40× ROIs classified within the low- and high-dMRI measure groups can be seen in the figures of the Appendix.

First, FA is known to be affected by the presence of crossing fibers, fiber dispersion, fiber bundle density, and by the microstructural architecture of the cellular membranes.^54^ For example, a 40× ROI containing the retrosplenial area and part of the cing was classified in the low-FA group. In this case, the low-FA value is due to the low-myelinated fiber density of this brain area. Indeed, the myelin fibers seen in this ROI have a smaller diameter and are not as tightly packed as the fibers in the fimbria near the septofimbrial nucleus, which was classified in the high-FA group. Second, low values of the NuFO measure are associated with fiber tracts and strongly aligned bundles, as shown here in the cc. On the contrary, high-NuFO values are measured in fiber crossings areas. This is observed for example in the gigantocellular reticular nucleus as shown in Fig. 10. Similarly, the AFD_max is another measure computed from the fODF and is interpreted as the AFD within a dMRI voxel. The OCM images classified within the low-AFD_max group show a low density of myelin fibers, as exemplified by the supplemental somatosensory area where the external capsule (ec) projects fibers in the cortex. On the opposite, high AFD_max is an indicator of high fiber density and was associated with the olfactory and temporal limbs of the anterior commissure (aco, act) in this example. The last two dMRI measures that were compared with the OCM data are OD index and the IC_VF from the NODDI model. Low OD is usually observed in the most coherent white matter as shown in Fig. 10 by the genu of the corpus callosum. As for the high-OD values, the original NODDI publication^44^ states that high OD is expected in “white matter structures composed of bending and fanning axons [and in] the cerebral cortex and subcortical gray matter structures characterized by sprawling dendritic processes in all directions.” For example, the caudoputamen near the ec was classified as having high OD. Finally, the intracellular volume fraction is an indication of the neurite density as it is related to the water diffusion constrained by the dendrites and neurons. To illustrate, an ROI containing the epithalamus and the dentate gyrus molecular and granule cell layers was classified within the low-IC_VF group, and an ROI containing four different fiber tracts (the superior cerebellar peduncle decussation, the mammilotegmental tract, the doral tegmental decussation, and the crossed tectospinal pathway) was classified within the high-IC_VF category. This qualitative comparison between the 2R-SOCT data and the dMRI measures shows that such an imaging pipeline will enable multiresolution and multimodal studies using small animal brains.

**Fig. 11:**
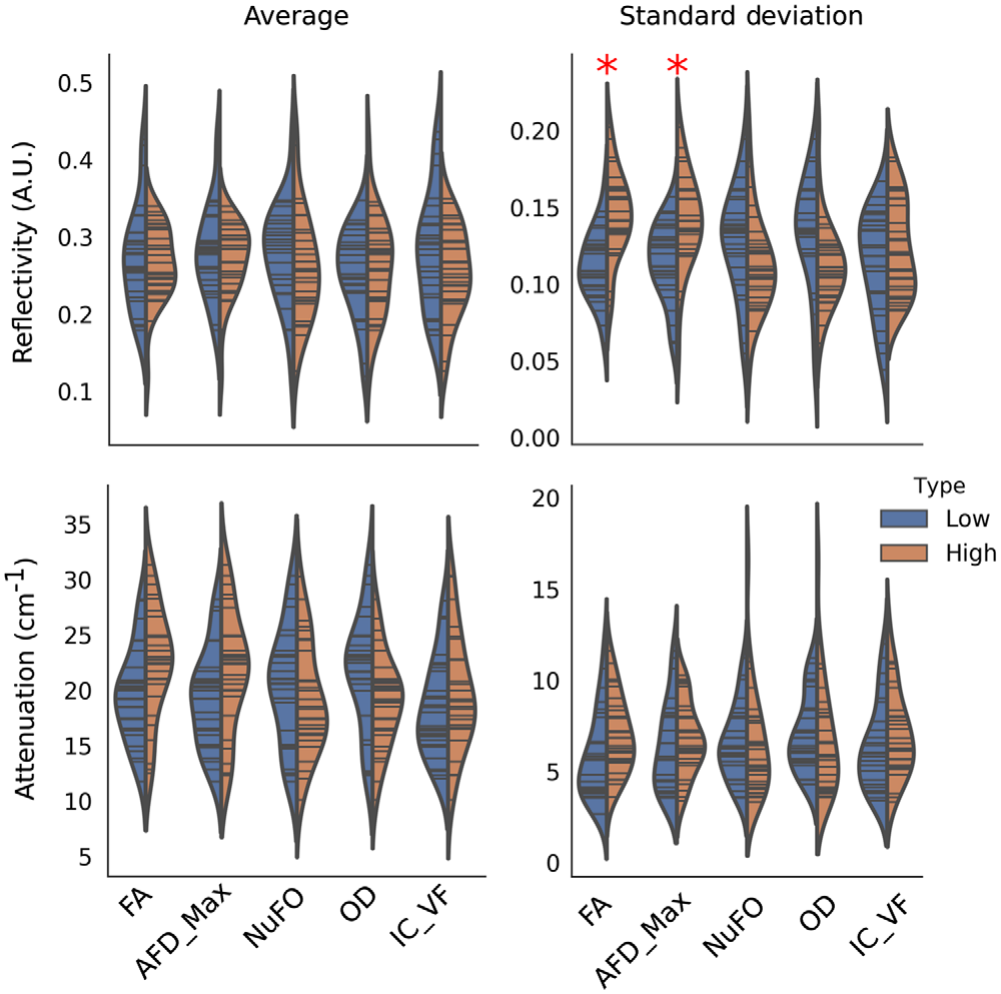
Comparison between simple OCM image features the dMRI metrics. Each histogram represents the OCM values classified within the low- (blue) and high- (orange) dMRI metric quantiles. The red stars indicate significant differences obtained from a T-test between all pairs of low-/high-metric values, corrected for multiple comparisons (p<0.0025).

A quantitative comparison between the OCM and the dMRI values was performed (Fig. 11). Image features were extracted from each 40× ROIs AIPs: the average normalized reflectivity $\left\langle r_{N}\right\rangle$, the average attenuation coefficient ⟨μ⟩, the normalized reflectivity standard deviation $\sigma_{r}$, and the attenuation coefficient standard deviation $\sigma_{\mu }$. The OCM image features associated with each 40× ROI containing at least one fiber tract structure were classified based on the dMRI metric groups computed for the qualitative comparison. For each feature ($\left\langle r_{N}\right\rangle$, ⟨μ⟩, $\sigma_{r}$, and $\sigma_{\mu }$) and for each pair of low-/high-dMRI metrics (FA, AFD_max, NuFO, OD, and IC_VF), a T-test was performed with a statistical significance level of α=0.05/n, where n=20 is the Bonferroni correction for multiple comparisons. This revealed that $\sigma_{r}$ was significantly higher for the ROIs classified within the low-/high-FA groups (p<0.00001), and for the ROIs classified within the low-/high-AFD_max groups (p<0.001). A hypothesis to explain this effect is that the presence of a fiber bundle within a 40× ROI increases tissue heterogeneity, thus broadening the OCT reflectivity value range. As the fiber bundle density increases, the overall reflectivity will increase in the FOV because more myelin fibers contribute to photon backscattering. The OCT signal for other dMRI metrics also seems to change between groups, although no statistically significant differences were measured. Some of the changes observed in Fig. 11 include an increase of ⟨μ⟩ (p=0.02) and $\sigma_{\mu }$ (p=0.05) with FA, as well as a reduction of $\sigma_{r}$ (p=0.02) and ⟨μ⟩ (p=0.04) with OD. A hypothesis to account for the higher attenuation with increases in FA is that as the fiber bundles become more strongly aligned, more myelin fibers are encountered by the photons and each reflection contributes to the sampling beam attenuation. This is consistent with our previous findings in whole mouse brains imaged with SOCT only.^9^ Furthermore, the reduction of $\sigma_{r}$ and ⟨μ⟩ as the neurite OD increases may be linked to myelin sheets and the anisotropic scatterers within the dendrites and axons that redirect the light in more directions, thus the overall reflectivity values range within the 40× ROIs decrease. This tissue thus appears as homogeneous. For gray matter, the neurites are less myelinated thus the tissue reflectivity will also decreases.

## 4 Discussion

The novelty of our proposed dual resolution serial OCT scanner lies in the fully automated acquisition procedure that allows accurate and repeatable positioning of high-resolution images within whole mouse brains. The two-objective configuration of the 2R-SOCT system benefits from the advantages of both types of OCT: the 3× OCT volumes are assembled into a single brain, which can be aligned to mouse brain templates and other imaging modalities, and the 40× OCT volumes are able to resolve individual myelinated fibers and neuronal cell bodies.^53^ Furthermore, the proposed system does not only work with dual-resolution OCT, but could also be adapted to other optical modalities. For example, using the lower resolution OCT to provide the stereotactic reference for a two-photon fluorescence laser scanning microscope. Another innovation of the 2R-SOCT technique is the automated acquisition procedure for the high-resolution images, which remove user bias in the selection of ROIs within the mouse brain. To our knowledge, the imaging pipeline presented in this paper is the first one to enable fully automated serial histology acquisitions at both low and high resolutions and the registration of the acquired data to a reference template. This allows to compare the high-resolution images to dMRI measures in a fully automated and repeatable way. We anticipate that this imaging technology will prove useful in multimodal MRI validation studies.

A few design choices of the 2R-SOCT system introduced limitations that could be improved in future implementations of similar serial microscopes. First, the same swept-source laser was used for both 3× and 40× arms. The narrow bandwidth (Δλ=100  nm) of the swept-source laser resulted in isotropic sampling for the 3× arm but in a high-sampling anisotropy between the axial and lateral directions for the 40× OCM volumes. This anisotropy limits the usefulness of the 3-D aspect of the 40× measures due to the poor axial resolution. Using a source with a larger bandwidth or distinct laser sources for each OCT arms could resolve this problem at the expense of increased cost and system complexity. Improving the axial resolution might also be possible by performing axial PSF deconvolution^55^ or interferometer synthetic aperture microscopy.^56^ Another imaging artefact caused by the optical design of the microscope is the focal plane curvature. This was characterized by detecting the water/tissue interface within each OCM volume and by next decomposing this surface into Zernike polynomials^57^^,^^58^ $Z_{j}(x,y)$ of index j≤5, thus only considering low-order aberrations. Typically, a focal plane depth denivelation of around 70 μm was measured between the center and the FOV boundary. For all OCM volumes, the field curvature geometry was near-spherical. Indeed, the three largest Zernike coefficients were, in decreasing order of their absolute normalized amplitude, the piston term $Z_{0}(|c_{0}|=0.5)$, the defocus term $Z_{4}$ ($|c_{4}|=0.26$), and the vertical astigmatism term $Z_{5}$ ($|c_{5}|=0.19$). The defocus term is a consequence of the large galvanometric mirror scanning angles necessary to get a lateral FOV of 0.5 mm with the 40× objective. The vertical astigmatism is caused by the two 45-deg mirrors used to guide the sampling beam toward the 40× arm and by the scan-induced delay.^59^ A different optical design, smaller lateral FOVs, the use of a lower magnitude objective, or an inline scan-induced delay compensation method could help to diminish this deformation.

Another design choice that increased the complexity of the system was the low-NA air-based 3× objective employed for the serial histology. An advantage of the 3× low-NA objective is the large depth-of-focus it provided (around 1.5-mm in air), which resulted in negligible lateral resolution variation with depth. Also the A-line axial range was large enough to be able to image both the current tissue slice and deeper areas corresponding to the couple next tissue slices. This provided information that can be used when performing axial coregistration between tissue slices for the whole brain reconstruction. A drawback of the air-based 3× objective was that a water-immersion chamber had to be added between the objectives and the sample to get a constant water thickness in the sample arm. During the acquisition, tissue debris and dirt accumulated on the optical window and resulted in a complex time-varying illumination inhomogeneity artifact. To address this limitation, one could replace the air objective by a water dipping low-NA objective. The time-varying artifacts could also be compensated in postprocessing, e.g., using an advanced retrospective background and shading correction algorithm.^60^

For this demonstration of the 2R-SOCT imaging pipeline, the dual resolution ROIs were either selected manually by the operator during the acquisition or in a fully automated way by employing an ROI selection algorithm. The manual selection method is useful to target specific brain areas and to investigate at high-resolution particular details observed during the serial histology acquisition. As for the automated ROI selection strategy, it offers the advantage of eliminating operator bias in the ROI positions, and it diminishes the total acquisition time as no user interaction is required, thus allowing for more dual-resolution ROIs to be acquired during an imaging session. Furthermore, the probability maps used to guide the random position selection can be adapted to use various image features and thus provide an additional degree of freedom during experimental design, such as local texture or the output of a multilayer convolutional network. In future work, the automatic ROI selection methodology could be combined with an *in situ* slice-to-volume registration procedure^61^ to an OCT mouse brain template. Using such a strategy could allow fully automated dual-resolution serial OCT protocols adapted to various experimental designs, such as automatic validation of dMRI in preselected fiber crossing areas, automatic multimodal studies with ROIs generated from user-defined segmentation of the OCT mouse brain template prior to the serial histology procedure, or acquisitions guided by previously computed statistical parametric maps obtained from another imaging modality. Other ROI selection strategies could be implemented to automatize the 2R-SOCT imaging pipeline, such as a regular grid, tissue segmentation into brain structures, and atlas-based selection with predefined probability maps.

Upon inspection of the brain structures associated with each ROI, it appears that each structure’s volume fraction is often lower than could be anticipated when observing the 40× ROI AIPs. For example, in Fig. 10, the ROI associated with a high-AFD_max value seems to mostly contain the olfactory limb of the aco, but this fiber tract only amounts to 29% of this ROI volume fraction. This can be explained as follows. First, the volume fractions were computed from the atlas labels contained within each 3-D ROI FOV coregistered to the Allen mouse brain template. Any registration mismatch can thus impact the structure volume fractions. The registration was performed with a series of rigid and affine transforms only to avoid the overfitting that can occur when using nonoptimal registration parameters. Thus smaller local morphological differences between the mouse brains and the template were not compensated. Another explanation for this volume fraction discrepancy is the image deformations and artefacts present in the 40× data. Indeed, the focal plane curvature, the presence of water above the tissue in the assembled OCM volumes, and the OCM reconstruction method itself can all reduce the effective axial FOV of the 40× ROIs, thus some structures that were intersected by the ideal 40× FOV might not be present in the effective FOV. Finally, this visual discrepancy between the reported volume fractions and the structures visible in the AIPs might only be a consequence of the 2-D representation of a 3-D FOV.

The larger size of the 40× ROI FOVs (0.5 mm) compared to the dMRI voxel size (0.125 mm) had for consequence that the average dMRI metrics per ROIs were affected by partial volume effects. For example, the maximum FA value measured for an ROI containing a fiber tract was 0.58. One option to reduce this effect in future investigations with the 2R-SOCT would be to split the ROIs into subvolumes of 125 μm prior to multimodal comparisons. A drawback of smaller ROIs is an increase sensitivity to data misalignment and registration errors, which in turn would increase the need to include nonlinear deformations into the multimodal registration pipeline. Finally, the naïve comparison of simple OCM image features with dMRI was shown to be limited. There was a lot of cross talks between each dMRI groups, and the statistical significance was low. The use of more complex image processing techniques, e.g., to extract information about the fiber volume fractions, fiber density, or orientation, could provide more information about the tissue microstructure in future dMRI validation studies with the 2R-SOCT platform. Nonetheless, the qualitative comparisons have shown that the 2R-SOCT imaging pipeline is able to target specific area in the brain and that the high-resolution ROIs can be located with good precision within diffusion MRI data.

## 5 Conclusion

A dual-resolution serial OCT imaging system was developed. The low-resolution OCT volumes acquired with a 3× objective were used to image whole mouse brains. The high-resolution OCM volumes acquired with the 40× objective were able to resolve individual myelinated fibers and other brain tissue structures. Moreover, the 2R-SOCT data were coregistered to dMRI data acquired for the same mouse brains prior to histology. This fully automated dual-resolution serial histology pipeline was used in a qualitative validation example, revealing the correspondence between various dMRI measures and fiber architecture. Our imaging pipeline demonstrates the usefulness of this imaging modality to perform multimodal validation studies and opens the way to interesting applications, such as small animal neuropathology multimodal cross-sectional studies. Finally, the 2R-SOCT imaging pipeline presented is not limited to OCT and OCM. Indeed, any imaging modality could be used in the second arm provided that the optical elements are adapted to the wavelength and type of signal to be measured. Such a dual-modality serial OCT scanner could be used, e.g., to evaluate colocalization in whole mouse brains of the microvasculature changes measured with a two-photon fluorescence microscopy, with the white matter distribution mapped with the SOCT.

## 6 Appendix

### 6.1 Compensation of the Time-Varying dMRI Signal

The fixed mouse brains were kept in their skull and stored in PFA at 4°C until the dMRI acquisition session. On the acquisition day, the sample to be imaged was removed from PFA and pat-dried, placed in a modified 10-ml syringe filled with Fomblin and placed in the dMRI machine. A sample was thus colder than room temperature at the beginning of the dMRI acquisition, and it reached temperature equilibrium during the imaging experiment. The sample temperature variation throughout the acquisition induced time-varying water diffusivity. This is a known effect associated with fixed sample imaging.^46^^,^^62^ To reduce the dMRI signal drift, the isotropic nature of diffusion in the cerebrospinal fluid (CSF) was exploited. First, the ventricles were segmented by thresholding the voxels in $b_{0}$ images based on their intensity. Then an average signal time profile was computed in the CSF by selecting 1000 voxels at random in the segmented ventricles. Assuming that the signal should be isotropic for each b-value in the CSF, the average signal was computed for each shell. A synthetic time profile was thus generated using the estimated average value for each shell. The signal drift was extracted by subtracting the synthetic multishell isotropic profile from the measured average time profile within the CSF. Finally, the signal drift was smoothed temporally using a Gaussian filter with σ=1 subscan. The time-varying signal was compensated by adding the computed drift bias to all DWI voxels.

### 6.2 Comparison Between 40× OCM and dMRI

**Fig. 12:**
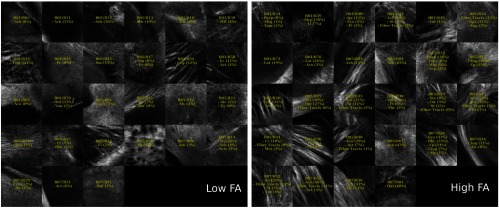
40× ROIs classified within the low- (<25% quantile) and high- (>75% quantile) FA groups. The yellow texts indicate the fiber tract structures within each ROIs, using the Allen mouse brain atlas structure acronyms.

**Fig. 13:**
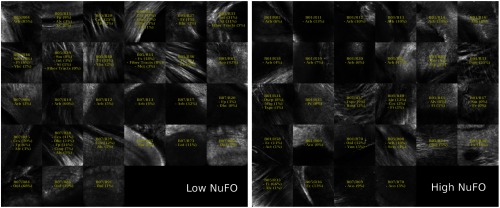
40× ROIs classified within the low- (<25% quantile) and high- (>75% quantile) NuFO groups. The yellow texts indicate the fiber tract structures within each ROIs, using the Allen mouse brain atlas structure acronyms.

**Fig. 14:**
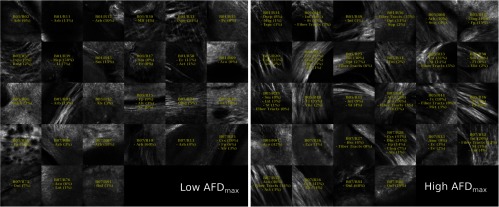
40× ROIs classified within the low- (<25% quantile) and high- (>75% quantile) AFD_max groups. The yellow texts indicate the fiber tract structures within each ROIs, using the Allen mouse brain atlas structure acronyms.

**Fig. 15:**
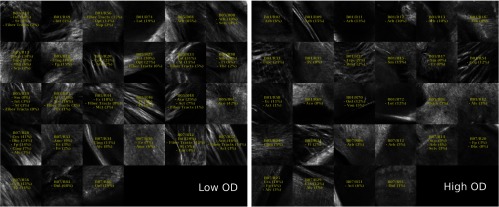
40× ROIs classified within the low- (<25% quantile) and high- (>75% quantile) OD index groups. The yellow texts indicate the fiber tract structures within each ROIs, using the Allen mouse brain atlas structure acronyms.

**Fig. 16:**
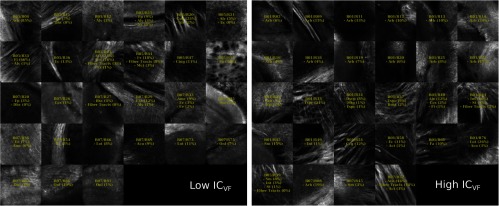
40× ROIs classified within the low- (<25% quantile) and high- (>75% quantile) IC_VF groups. The yellow texts indicate the fiber tract structures within each ROIs, using the Allen mouse brain atlas structure acronyms.

The multimodal registration results were evaluated visually by comparing the aligned whole mouse brains with the dMRI data and by comparing the identified structures within the 40× dMRI with those extracted from the Allen mouse brain atlas using only the ROI positions obtained from the coregistration. As seen in Figs. 12–16, the same structures were seen in the 40× ROIs as predicted by their positions within the Allen mouse brain atlas after registration, which indicates that the registration errors were reasonably low. Further investigation would be required to quantitatively assess the registration errors introduced by the multimodal registration procedure and inherent to the 2R-SOCT acquisition. This would require a much higher dMRI resolution in selected ROIs to enable an additional direct registration between the OCM and dMRI. This cannot be achieved with the existing data as the dMRI data resolution was 125 μm, which represents a quarter of the 40× OCM FOV size and renders all fine coregistration attempts futile.

Upon visual inspection of the OCM images classified in the low-/high-dMRI metric groups (Figs. 12–16), one can notice that the FA/AFD_max and the NuFO/OD metrics seem to originate from tissue areas characterized by similar fiber architectures. These dMRI measures are all based on different hypotheses and they are interpreted in different ways when used in dMRI studies. The fact that some dMRI metrics seem to be correlated when visualizing the OCM ROIs is an interesting result. Analyzing the OCT fiber architecture with more advanced image processing techniques and comparing the results with various dMRI models could help validate the hypothesis of each model, or it could indicate that some dMRI models have erroneous hypothesis or are wrongfully interpreted.
